# Mindfulness, Interoception, and the Body: A Contemporary Perspective

**DOI:** 10.3389/fpsyg.2019.02012

**Published:** 2019-09-13

**Authors:** Jonathan Gibson

**Affiliations:** Department of Humanities and Social Sciences, South Dakota School of Mines and Technology, Rapid City, SD, United States

**Keywords:** mindfulness, interoception, meditation, yoga, body-awareness, contemplative practice, mind-body

## Abstract

Mindfulness is often used as an umbrella term to characterize a large number of practices, processes, and characteristics. Critics argue that this broad definition has led to misinformation, misunderstanding, and a general lack of methodologically rigorous research. Some of the confusion surrounding mindfulness is also believed to stem from an undifferentiated use of the term mindfulness and meditation. Mindfulness and all other forms of meditation have been shown to modulate the insula, which is the primary hub for interoception. Some have argued that interoception is foundational to mindfulness and may be the primary mechanism by which one benefits from the practice. However, much like the mindfulness literature, interoception remains broadly defined often without precision and with domain-specific meanings and implications. Research demonstrates that the insula and surrounding neural circuits are believed to be responsible for a number of other functions beyond interoception including attention, awareness, and all subjective experiences, much of which has been linked to the mindfulness literature. It has been assumed that mindfulness produces these neuroplasticity and functional effects. There is evidence that mindfulness and some of its benefits may be better described as increased interoception as a result of the neuroplasticity changes in the insula, and the development of the insula and surrounding neural circuits may cultivate dispositional mindfulness. The purposes of this article are to (1) highlight that it may be more accurate to link many of the identified benefits in the mindfulness literature to interoception and its neurological correlates and (2) propose attentional style as a means to clarify some of the confusion surrounding mindfulness, interoception, and meditation. Different meditations require different attentional styles. Attention can be analogous to a focal point with each focal point providing a unique perspective. Given that all meditative techniques modulate the insula, each meditation can provide a unique perspective from which to investigate complex interoceptive signals that may be unavailable from other meditative traditions. It may prove more useful to anchor scientific findings in the concrete body as a means to investigate those rather than a set of abstract, broadly defined meditative techniques.

## Introduction: Mindfulness Broadly Conceptualized

Mindfulness has grown in popularity in the scientific literature over the past two decades. Despite many of the documented benefits, mindfulness has come under some scrutiny as of late. One of the challenges in the mindfulness literature is how to define it. Mindfulness is often used as an umbrella term to characterize a large number of practices, processes, and characteristics (Van Dam et al., 2017). Though mindfulness has its roots in Buddhism, it has extended beyond that tradition into the sciences and Western contemporary culture. Grossman (2019) argues that this can be problematic because mindfulness practice in Buddhism is meant to cultivate “truths” about personal, lived experiences – a subjective phenomenon that is difficult to measure with empirical investigation. Furthermore, the definition and measurement of mindfulness in modern science are enmeshed in a complex web of historical, social, economic, political, and technological factors (Grossman, 2019). Van Dam et al. (2017) point out that in this expansion, mindfulness has been defined in a variety of ways, which has led to misinformation, misunderstanding, and a general lack of methodologically rigorous research.

Some of the confusion surrounding mindfulness is believed to stem from an undifferentiated use of the term mindfulness and meditation. There is no universally accepted technical definition of “mindfulness” nor is there a consensus on various aspects underlying the concept to which it refers (Van Dam et al., 2017). For instance, mindfulness has been described as a mental faculty for being consciously aware or present in a given moment and taking account of the currently prevailing situations (Kabat-Zinn, 1990). Mindfulness may also refer to a particular meditation – whether it is an open-monitoring meditation, breathing mediation, or body scan (Van Dam et al., 2017). Mindfulness has been conceptualized as a mental faculty relating to attention, awareness, memory, or discernment (Davidson and Kaszniak, 2015). Mindfulness has also been envisioned as a path to psychological and physical well-being (Grossman, 2019). The semantic ambiguity in the meaning of mindfulness has implications. Van Dam and his colleagues argue that any study using the term mindfulness must be carefully scrutinized to accurately ascertain what type of “mindfulness” was involved. When formal meditation is used in a study, one ought to consider whether a mindfulness or another meditation was the target intervention.

There are also a variety of methodological challenges that face mindfulness studies. For a review of some of those, see Van Dam et al. (2017) and Grossman (2019). In this article, I will examine some of the possible effects of the undifferentiated use of the term mindfulness and meditation and the implications that may stem from those. Van Dam et al. (2017) urged practitioners, scientists, and the public news media to move away from the broad umbrella term of mindfulness to more explicit and differentiated construals of exactly what practices and processes are being taught. It may be in the imprecision of the definition and a subtle lack of understanding of these various constructs that have left ambiguous and conflicting results. Indeed, recent meta-analyses on various meditations highlight that different meditations produce different effects and engender different results both neurologically and psychologically (Fox et al., 2014, 2016; Fox and Cahn, 2017).

In popular culture, there are a variety of definitions, facets, or concepts that circumscribe mindfulness. In the scientific literature, several definitions have been proposed. Kabat-Zinn (2003) defined mindfulness as the awareness that emerges through paying attention on purpose, in the present moment, and non-judgmentally to the unfolding of experience, which includes sensations, cognitions, and emotions, moment by moment. A more recent definition includes moment-to-moment awareness, cultivated by paying attention in a specific way, in the present moment, as non-reactivity, non-judgmentally, and open-heartedly as possible (Kabat-Zinn, 2011). Brown and Ryan (2003) suggested that attention to the moment is the single most critical aspect of mindfulness, supplanting acceptance and non-reactivity. Shapiro et al. (2006) posit that attention, intention, and attitude are the three most important components of mindfulness, and paying attention to one’s experience can lead to changes in perspective, what has been referred to as decentering. Some of the key components of mindfulness that consistently emerge in the scientific literature include present-moment awareness, beginner’s mind, non-judgmental attitude, non-reactive, and acceptance (Mehling et al., 2017). It is important to note, however, how mindfulness is operationalized will determine what is measured and how – which can differ from scale to scale (Grossman, 2019).

## Mindfulness Findings

Kabat-Zinn (1990) argued that mindfulness can be developed in formal meditation practices such as sitting meditation, walking meditation, or mindful movements. More recent research has shown that facets of mindfulness can be cultivated by a variety of contemplative practices, whether it is a focused attention (FA) meditation such as a breathing meditation (Gibson, 2014), an open-monitoring (OM) meditation often referred to a mindfulness meditation that consists of paying attention to internal and external sensations in an open, accepting, and non-judgmental way (Raffone and Srinivasan, 2010), or yoga-based practices (YBPs; Villemure et al., 2014; Schmalzl et al., 2015). It is argued that purposefully focusing attention on internal sensations cultivates non-judgmental moment-to-moment awareness that permeates into daily life (Kabat-Zinn, 1990; Silverstein et al., 2011). When engaging in a mindfulness meditation, the individual’s goal is to maintain attention on internal or external stimuli with a non-judgmental stance, manifesting acceptance, curiosity, and openness (Hölzel et al., 2011).

Mindfulness and other meditations have consistently been associated with improvements including, but certainly not limited to, cognitive performance, attentional capacities, negative emotional states associated with self-judgment, increased compassion and prosocial behavior, reduced stress, improvements in depression, anxiety, and other clinical conditions (Shahar et al., 2010; Silverstein et al., 2011; Van Dam et al., 2011; Hanley et al., 2017; Yeng et al., 2018). Yet, as some critics point out, mindfulness training programs do not always reveal improvements on mindfulness scales (Witek-Janusek et al., 2008; Barrett et al., 2012), and when scores differ from controls, the effect sizes can be modest or even weak (Quaglia et al., 2016; Spijkerman et al., 2016). Due to the multi-dimensionality of mindfulness as construct and inconsistent results in the literature (see Van Dam et al., 2017 and Grossman, 2019 for a review), Grossman (2019) argues that science should currently be aimed at investigating the constituent elements identified in the literature instead of mindfulness as the gateway to those constituents. Hölzel et al. (2011) identified four constituent elements that consistently emerge, which include (1) attention regulation, (2) body awareness, (3) emotion regulation, and (4) change in perspective on the self.

Another important constituent that has emerged in the literature is that mindfulness and other meditations improve internal awareness and that developed capacity has led to increased clarity of mental processes leading to improvements in meta-awareness (Cahn and Polich, 2006; MacLean et al., 2010; Raffone and Srinivasan, 2010; Schmalzl et al., 2015). Meta-awareness has been described as a form of subjective experience and executive monitoring that is not entangled in the contents of awareness but rather facilitates a detachment form of identification with the sense of a static or unchanging self (Hölzel et al., 2011). The self, in this state, can be experienced as an event rather than a static entity (Olendzki, 2006). Some have argued that mindfulness can cultivate a detachment or decentering that supports a decoupling of habituated reactions, behaviors, or thought patterns (Carmody et al., 2009; Hölzel et al., 2011) and can facilitate self-regulation allowing the person to focus on the present moment (Chambers et al., 2009). This decoupling allows for a change in the perspective of the self. According to Buddhist philosophy, a change in the perspective of the self is central to developing mindfulness. Neuroimaging studies have shown that mindfulness meditations produce neuroplasticity changes in the brain, which appear to support those functional changes. Mindfulness has been shown to produce neuroplasticity effects in a number of different brain regions, but relevant to this article are the changes found in the default mode network (DMN; Hölzel et al., 2011) and the interoceptive network that spans various brain regions, which include the insular cortex, cingulate cortex, inferior frontal gyrus, and the sensorimotor cortex (Craig, 2002, 2009; Critchley et al., 2004; Farb et al., 2007, 2013; Friedel et al., 2015; García-Cordero et al., 2016; Haase et al., 2016; Pollatos et al., 2016).

## Interoception: Historical and Contemporary Conceptualization

Interoception or interoceptive awareness (IA) is believed to be a key component to many mindfulness interventions. Some have argued that interoception is foundational to mindfulness and may be the primary mechanism by which practitioners derive benefits from the practice (Mehling et al., 2012; Farb et al., 2013, 2015). Most mindfulness trainings or mindfulness-based interventions (Mindfulness-Based Stress Reduction – MBSR, for example) are believed to cultivate IA resulting in a shift away from thinking about body sensations to an immediate feeling of body sensations (Farb et al., 2013, 2015; Mehling et al., 2017). Both mindfulness and IA are tightly interwoven yet distinct constructs and appear to be independently associated with enhanced psychological well-being (Hanley et al., 2017).

Research on interoception or IA has spanned for more than a century, and the findings, historically, have been nebulous. Indeed, much of the research has led to vary understandings and definitions (Cameron, 2001; Mehling et al., 2009). Interoception has gained more recent attention in the academic literature; however, much like the mindfulness literature, the construct itself remains broadly defined often without precision and with domain-specific meanings and implications (Mehling et al., 2009, 2012; Farb et al., 2015; Ceunen et al., 2016). Most of the literature currently supports a working definition of IA to include a perception of the internal state of the body. This contemporary definition includes sensory awareness that originates from within the body’s physiological state as well as a person’s appraisal, attitude, belief, past experiences, expectations, and contexts, what is often referred to as top-down perceptions (Cameron, 2001; Craig, 2009; Mehling et al., 2012; Farb et al., 2015) providing moment-by-moment representation of the body’s internal milieu (Craig, 2009; Khalsa et al., 2018). Even with this recent shift in defining and conceptualizing interoception and a focused effort to develop a common taxonomy, it still remains poorly understood in modern science (Farb et al., 2015), and there is no consensus on its meaning (Ceunen et al., 2016). Khalsa et al. (2018) argue that interoception lacks a coherent integration across all implicated bodily systems, and it has been questioned whether interoception or interoceptive awareness is too broad to encompass any or all the various dimensions available for self-report.

One problem as Mehling et al. (2009) point out is that terms, such as body awareness or somatic awareness, are often used interchangeably with interoception or interoceptive awareness. These constructs are typically operationalized in a similar manner – awareness of the internal bodily state – which can be problematic because there is conflicting, even contradictory evidence as to the benefits and detriments of attending to one’s body as identified in the IA and the body awareness (BA) literature. For instance, the BA literature, which is slanted toward a clinical population, suggests that heightened awareness of bodily sensations often serves as markers for a variety of mental disorders including anxiety, panic disorder, depression, eating disorders, somatization, and others (Cioffi, 1991; Pollatos et al., 2008, 2009; Mehling et al., 2009; Herbert and Pollatos, 2014; Mallorquí-Bagué et al., 2014; Fischer et al., 2017). Therefore, it is assumed that increased awareness to bodily sensations can be distressing and maladaptive (Baas et al., 2004; Mehling et al., 2009). Conversely, much of the IA research has found a positive relationship between the degree of IA and emotional regulation (Wiens, 2005; Dunn et al., 2007; Herbert et al., 2011), decision making (Dunn et al., 2012; Sütterlin et al., 2013), empathy (Singer et al., 2009; Fukushima et al., 2011), behavioral regulation (Herbert et al., 2007, 2012; Herbert and Pollatos, 2014), and others. Maladaptive forms of IA seem to be characterized by hypervigilance and catastrophizing, while healthy forms are characterized by attention regulation and acceptance (Hanley et al., 2017). These conflicting findings indicate that IA is a complex multi-dimensional construct (Mehling et al., 2012). Interoception seems to require the interplay between perceptual body states and cognitive appraisal of those body states (Farb et al., 2015).

Some of the conflicting evidence in the literature may stem from the limited historical definitions and methodologies. For instance, Dworkin (2000) defined IA as the perception of afferent information from receptors that monitor the internal state of the body in which he distinguishes the differences from interoceptors (blood vessels, CNS, and visceral organs), proprioceptors (skeletal and joint receptors), and exteroceptors (photoreceptors of the eye and mechanoreceptors of the skin). Other studies argued that IA is a stable characteristic similar to vision. That is, some either have good IA (like good vision), and the others have poor IA (like poor vision; Ehlers, 1990; Ehlers and Breuer, 1996). Furthermore, most of the IA and BA literature prior to 2012 relied on deductive experimental methodologies, which assumed that this ability can be quantified and measured within a single modality, i.e., measuring visceral signals. However, studies investigating IA across different visceral modalities (i.e., heartbeat detection, gastrointestinal activity) in healthy persons under controlled conditions were sparse, and findings were typically ambivalent (Steptoe and Noll, 1997; Herbert et al., 2012). Some psychopathologies are quite mixed in IA capacity. For instance, participants with both panic disorder and eating disorders have shown an enhanced ability to detect cardiovascular signals (Ehlers and Steil, 1995), while the other studies found no reliable differences (Barsky et al., 1994; Van der Does et al., 2000).

In an effort to try to further clarify interoception or IA, several more nuanced and sophisticated models have recently emerged. Garfinkel et al. (2015) presented a three-dimensional model that differentiates between interoceptive accuracy (IAc), interoceptive sensibility (IS), and interoceptive awareness (IA). Interoceptive accuracy (IAc) has been proposed as the ability to accurately detect signals from within the body and is believed that this capacity can be objectively measured. The preferred method to measure the objective dimension of interoception or IAc has been the heartbeat detection (HBD) task (Schandry, 1981). There are other physiological tests that have been used such as the water load test based on the sensation of gastrointestinal signals (Herbert et al., 2012). Though the HBD task has come under some scrutiny as of late (Brener and Ring, 2016), the HBD task is often considered the standard and preferred method to objectively measure interoception or IAc (see Critchley et al., 2004; Dunn et al., 2007; Füstös et al., 2012; Bornemann et al., 2015; Fischer et al., 2017). Interoceptive sensibility (IS) is described as a second dimension in interoceptive processes by the evaluation or subjective confidence in accurately identifying the internal bodily state. This capacity is measured by subjective self-report questionnaires (see, for example, the Body Perception Questionnaire, Porges, 1993; the Multidimensional Assessment of Interoceptive Awareness – MAIA, Mehling et al., 2012). Currently, it is generally accepted that the subjective element of IA is influenced by top-down cognitive processes such as memories, expectations, attitudes, and changes in context (Mehling et al., 2012; Bornemann et al., 2015; Farb et al., 2015). The third dimension in Garfinkel et al.’s model is a meta-cognitive dimension or what the authors term interoceptive awareness (IA). In this dimension, IA combines both IAc and IS or the objective and subjective measures. The goal of IA, ultimately in this model, is to analyze the accordance between confidence and accuracy of interoceptive abilities (see also Fischer et al., 2017).

Mehling et al. (2012) advanced another model arguing that interoception is a multidimensional construct, and this self-report measure includes eight different dimensions of IA. One of those dimensions, noticing, is believed to capture bodily physiological sensations such as heartbeat or breath. This is similar to Garfinkel et al.’s (2015) model capturing IAc. The other seven dimensions include regulatory and attentional aspects of body awareness that are believed to reflect IS. That is, how the body and its felt sensations are internally used by the subject to regulate stress or attention and how those sensations are linked to one’s emotional state to the extent to which the body is experienced as safe or discomforting (see also Mehling, 2016). It is also important to note that interoceptive signals do not need to reach conscious awareness in order to influence one’s psychological state. When interoceptive sensations do reach conscious awareness, those seem to have a greater effect on cognitions, emotions, perceptions, and behavior (Cameron, 2001; Dunn et al., 2007; Craig, 2009).

It has been assumed that IA could be measured and quantified by an objective test. The HBD task is believed to be a reliable and valid quantifiable measure of IA (Whitehead and Drescher, 1980; Schandry, 1981), in which the participants are instructed to perceive his or her heartbeats without feeling for a pulse. Although HBD can be reliably detected, the assumption that HBD serves as an indicator of “general” IA for bodily signals is still unresolved (Herbert et al., 2012; Ferentzi et al., 2019). Some critics argue that the reason to use the HBD method over other methods and measures is because it can be easily measured and is assumed to reflect a general sensitivity to visceral processes (Bogaerts et al., 2008). However, some bodily signals such as heartbeats are more easily perceived than other visceral signals (Kollenbaum et al., 1996).

Recent studies indicate that objective interoceptive measures may only partially overlap, and different sensations and body systems seem to elicit distinct neural processes (Baranauskas et al., 2017; Yeng et al., 2018). Given that interoception comprises multiple systems that present many challenges on how to measure those. One approach is to measure multiple organ systems simultaneously or to record activity in the peripheral organs, spinal cord, and brain (Khalsa et al., 2018). The complexity of both the physiological processes and the perception of those raises the question: can HDB serve as a generic indicator of IA or at least the objective dimension (IAc) of IA? The results are mixed, and the evidence is limited.

Some recent studies have found minor improvements in HBD in longitudinal interoceptive training practices (Bornemann and Singer, 2016; Fischer et al., 2017; Garcia-Cordero et al., 2017). Most studies, however, found no improvement in the HBD task (Nielsen and Kaszniak, 2006; Khalsa et al., 2008; Melloni et al., 2013; Parkin et al., 2013, 2014; Bornemann et al., 2015). There are several possible explanations for this. As Bornemann and Singer (2016) point out, most studies that failed to improve HBD employed cross-sectional designs with a small *n* size. The practiced meditators in those studies came from diverse backgrounds (i.e., Buddhist or Kundalini traditions), few studies employed longitudinal designs, and most did not focus solely on the single effect of a body-centered practice such as a body scan meditation on the objective measure of IA. There were two studies that have employed longitudinal designs on HBD, and both found conflicting results. One group (Fischer et al., 2017) showed modest improvement in accurately detecting HBD in a longitudinal study, while the other did not (Parkin et al., 2014). García-Cordero et al. (2016) argue that neuroimaging results suggest that improved interoceptive accuracy – what they termed interoceptive learning – seems to rely on neuroplasticity changes due to interoceptive training which can include mindfulness and other mind-body contemplative practices.

Interestingly, Garcia-Cordero et al. (2017) conducted another study investigating heartbeat accuracy and found that when participants focused on exteroceptive stimuli, not interoceptive sensations, their performance improved. The authors argue that interoception and exteroception may use different neural mechanisms and that exteroception may involve less demanding perceptual operations with subjects relying on well-developed sensory skills. Interoception may be more cognitively demanding as attention must be directed inward and rely on less trained or developed mechanisms. To complicate matters further is that the somatosensory cortex, which is involved in processing exteroceptive stimuli, is generally included as a part of the interoceptive network (Farb et al., 2007, 2013).

Another explanation why studies investigating the effect of contemplative practices on objective measures of IA are so inconsistent is that the interoceptive network was found to be insufficient for awareness of HBD (Khalsa et al., 2009). Instead, the authors found that HBD was mediated by two pathways; somatosensory afferents from the skin and the interoceptive network that included the insula and ACC. “Together, these pathways enable the core human experience of the cardiovascular state of the body” (p. 1494). In that study, once the participant’s chests were anesthetized, they were no longer able to detect their heartbeat at rest (Khalsa et al., 2009).

It is generally believed that to improve interoception relies on attention and perception of internal stimuli (Mehling et al., 2012; Bornemann et al., 2015; Garcia-Cordero et al., 2017). Interoceptive learning appears to be a complex process that includes updating and integrating information from current body signals with previous body signals and mental models (Craig, 2009; Mehling et al., 2012; Farb et al., 2013). Over time, these connections are strengthened. The brain’s interoceptive system may be a necessary but insufficient condition for HBD. However, this network is believed to be implicated in the cognitive improvements and emotional regulation that are linked to increased IA (Farb et al., 2007, 2013; Craig, 2009; Dreeben et al., 2013; Bornemann et al., 2015; Fischer et al., 2017). It could be plausible that contemplative practices, including mindfulness, may only affect certain dimensions related to IA as it is studied in both the objective and subjective measures. Indeed, there is growing evidence that a variety of contemplative practices such as meditation (Lazar et al., 2005; Hölzel et al., 2008; Kang et al., 2013), mindfulness (Farb et al., 2007, 2013, 2015; Haase et al., 2016), yoga (Villemure et al., 2014; Schmalzl et al., 2015), tai chi (Kerr et al., 2008), and likely other mind-body practices enhances and improves IA. How to objectively and subjectively measure that is ongoing.

One study found no noticeable changes in an objective or “noticing” dimension as measured by the MAIA after three months of interoceptive training (Bornemann et al., 2015). There were, however, substantial changes in the subjective, self-regulation, and attentional dimensions. The authors argue that participants *via* interoceptive training (body scan meditation and breath meditation) learned to redirect attention to their body, which helped them manage stress, regulate emotion, and facilitate cognitive insight derived from their body/emotional state. Two studies investigating the impact of mindfulness-based interventions designed to increase interoceptive awareness found similar results (de Jong et al., 2016; Fissler et al., 2016). Not surprisingly, those quantitative findings reflect many descriptive reports of participants practicing various contemplative, mind-body practices (Landsman-Dijkstra et al., 2004; Morone et al., 2008; Schure et al., 2008; Gibson, 2014). Paulus and Stein (2010) concluded that IA cannot be sufficiently understood by objective interoceptive signals alone, but those signals are part of a larger self-referential and predictive belief state.

The complex multi-dimensionality of interoception, which includes regulatory aspects and attentional styles, does not easily map onto a single-objective measure (Mehling, 2016). Indeed, the HBD task has received some criticism recently and is not considered to be a reliable indicator of cardioceptive accuracy (Brener and Ring, 2016). It has also been argued that non-pathological interoceptive information is usually ambiguous; thus, perception of interoceptive stimuli is often influenced by top-down perceptions (Brown, 2004; Mehling et al., 2012; Farb et al., 2015). Ferentzi et al. (2017, 2018) found that the objective measure of interoception (IAc) cannot be generalized across interoceptive modalities. A recent study has found that subjective well-being is only correlated to the subjective domains of interoception (Ferentzi et al., 2019). That is, well-being is only associated with perceived subjective dimension of enhanced interoceptive awareness but not related to the objective, sensory measures (i.e., HBD or gastric signals) of interoceptive accuracy (IAc).

It may be that interoception or interoceptive awareness can no longer be considered equivalent to interoceptive accuracy (Mehling, 2016). There seems to be considerable dissociation between perceived and actual body-related events (Ferentzi et al., 2017, 2019). As Fischer et al. (2017) point out, most studies show no difference between intensive mindfulness programs (MBSR, for example) and isolated performance of the body scan meditation to improve IA as measured by objective measures. It is reasonable to conclude that interoceptive awareness may be only one aspect of interoceptive processing. Interrelated but distinct constructs may include interoceptive sensitivity, attention tendency, coherence between actual physiology and subjective experience, and interoceptive accuracy (Farb et al., 2015). Interoception may include the act of sensing, interpreting, and integrating information about the state of the inner body, and this process can be related to interoceptive attention, detection, discrimination, accuracy, insight, sensibility, and self-report with much of these processes occurring below conscious awareness (Khalsa et al., 2018).

In most studies, and consistent with the assumptions from many researchers in the field, mindfulness and other contemplative practices do lead to improvements in the subjective dimensions of interoception as measured by self-report measures such as the MAIA (Mehling et al., 2012), qualitative descriptions (Landsman-Dijkstra et al., 2004; Morone et al., 2008; Schure et al., 2008; Gibson, 2014), and neuroplasticity changes in the interoceptive network (Farb et al., 2007, 2013; Friedel et al., 2015; García-Cordero et al., 2016; Haase et al., 2016; Pollatos et al., 2016) but have found inconsistent results in measuring IA in the objective domains. The differences between objective and subjective domains may be due to strengthening cognitive and affective processes, especially evaluations, memories, attitudes, which may be distinct from measurable, objective interoceptive processes (Parkin et al., 2014). This finding ultimately raises the question: Does the objective dimensions of interoception measured by HBD or gastric signals inform or influence the subjective self-regulatory and attentional domains of IA? Does increased sensitivity and awareness to one’s own heartbeat help regulate emotion and enhance cognitive performance? Attentional style or how one responds to those sensations may be the lynchpin to those questions.

Ferentzi et al. (2019) concluded that interoceptive accuracy (IAc) does not have a direct positive or negative impact on well-being, rather well-being depends more on subjective interpretation of those sensations. Mallorquí-Bagué et al. (2014) demonstrated that higher anxiety was associated with higher interoceptive accuracy as measured by the heartbeat tracking task. Mehling (2016) found that the attention style toward body symptoms, specifically attentional and regulatory dimensions of IA, is negatively correlated with anxiety. Mehling (2016) identified an interesting pattern between emotional susceptibility and interoceptive sensibility (IS). Participants with higher emotional susceptibility often report more emotional distress or worry with sensations of discomfort or pain. For some, especially in a clinical population, increasing interoceptive awareness without the ability to use awareness of those sensations may actually increase anxiety. Conversely, attending to those sensations in a self-regulatory or mindful way appears to be adaptive.

## Mindfulness and Different Meditation Types

It is important to be precise and clear how we define and circumscribe mindfulness and meditation (Van Dam et al., 2017). An essential component in both mindfulness and other meditative practices is learning how to intentionally focus on body sensations and redirect it when the mind has wandered. Meditation comprises a number of different techniques, but some have grouped these techniques into a few broad categories. One model identifies two basic meditative categories: focused attention (FA) and open monitoring (OM; Lutz et al., 2008; Raffone and Srinivasan, 2010). Focused attention (FA) involves focusing and maintaining attention on a single object such as one’s breath, heartbeat, or a mantra. Open monitoring (OM) meditation is often referred to as a mindfulness meditation, which involves a non-reactive, non-judgmental monitoring of ongoing experiences that includes thoughts, internal sensations, and external stimuli (Raffone and Srinivasan, 2010; Fissler et al., 2016). Focused attention meditations have shown to help regulate attention that includes the enhanced ability to detect and disengage from a distraction and refocus attention (Lutz et al., 2008; Raffone and Srinivasan, 2010; Schmalzl et al., 2015). It is also believed that FA meditations can lead to an enhanced ability to decrease mind-wandering and increase attentional stability (Lutz et al., 2008; Farb et al., 2015).

Open monitoring (OM) meditation or mindfulness meditation consists of a broad category of practices and does not involve specific attentional focus per se, but rather is characterized by an open-presence and non-judgmental awareness of sensory, cognitive, and affective domains in the present moment (Cahn and Polich, 2006). Open monitoring is also believed to involve higher-order meta-awareness (MacLean et al., 2010; Schmalzl et al., 2015) enabling an objective witnessing of one’s stream of thoughts and experiences. To be able to witness one’s experience without judging them is believed to lead to a gradual lessening of one’s identification with those thoughts (Schmalzl et al., 2015). In some mindfulness traditions, there is a gradual shift over time from FA to OM techniques. One may begin to focus his or her meditation on the breath or bodily sensations and gradually, over time, transition to a more open presence noticing all sensory, emotional, and cognitive processes (Raffone and Srinivasan, 2010; Fissler et al., 2016). One prominent Buddhist scholar argued that attentional stability is necessary for the cultivation of contemplative insight (Wallace, 1999). Farb et al. (2015) posit that with the stability of interoceptive anchoring, practitioners can engage in an OM form of attention that may reveal or weaken habitual patterns. The skills further developed and honed by FA meditations seem to enhance the effectiveness of OM or mindfulness meditations.

Each of these categories of meditation has been studied from a neuroimaging perspective. It is now widely accepted that meditation promotes anatomical (Lazar et al., 2005; Hölzel et al., 2008; Farb et al., 2013; Kang et al., 2013; Fox et al., 2014) and functional brain changes (Farb et al., 2007, 2013; Zeidan et al., 2011; Tang et al., 2012; Fox and Cahn, 2017). A recent meta-analysis investigating the functional and anatomic differences each meditation produces highlights, “psychologically distinct meditation practices show correspondingly diverse neural correlates” (Fox and Cahn, 2017 p. 13). Even with all the differences, both psychologically and neurologically, between the various meditative practices, there are relevant similarities important to note in this article. Foremost among those similarities is that all types of meditations, i.e., FA, OM, and even loving/kindness have been found to modulate the insula in some way (Fox and Cahn, 2017). Given the insula’s role in interoception and the central role of the body in mindfulness and other contemplative practices, this finding should not be surprising (Fox and Cahn, 2017). Indeed, a number of studies have highlighted that mindfulness meditation specifically modulates the insula and interoceptive network (Farb et al., 2007, 2013; Paulus et al., 2011; Santarnecchi et al., 2014; Friedel et al., 2015; Haase et al., 2016).

## Why This Matters: Functions of the Insula and the Interoceptive Network

A number of Eastern traditions recognized the importance of interoception and the body and developed a variety of contemplative practices devised to cultivate IA. The concept that the body and IA play a central role in psychological processes – that the body influences brain function – is not new in Western science either but has been argued since the nineteenth century (Darwin, 1872; James, 1894; Bernard, 1973). Early and modern theories of emotion and consciousness have emphasized the importance of interoceptive feedback in emotional states and cognitive processes (James, 1894; Damasio, 1994; Craig, 2002, 2009; Porges, 2007, 2011; Mayer, 2011). For example, the somatic marker hypothesis advanced by Damasio (1994) argues that positive or negative emotional feeling states are associated with visceral and other bodily responses to certain situations. Damasio et al. (1996) posit that IA is essential for most affective, cognitive, and interpersonal processes. This capacity supports our sense of self and is crucial for relating to the outer world (Damasio, 2003).

Extensive evidence shows that the image of the body’s internal state is ultimately represented in the insula and interoceptive network (see Craig, 2002, 2009 for review). The interoceptive network spans various brain regions, which include the insular cortex, cingulate cortex, the inferior frontal gyrus, and the sensorimotor cortex (Craig, 2002, 2009; Critchley et al., 2004; Pollatos et al., 2016; Garcia-Cordero et al., 2017). This network also presents multiple connections to the amygdala, hypothalamus, hippocampus, and brainstem (Craig, 2009; Becker et al., 2015; Kleint et al., 2015; Khalsa et al., 2018). The insula is believed to be the key region, which integrates information from the body *via* lamina 1 spinothalamic and vagal afferent tracts (Craig, 2002). Much of these body sensations projects ultimately into the posterior portion of the insula and somatosensory cortices and is re-represented in the mid and anterior (aINS) portion of the insula (Craig, 2009; Farb et al., 2013). The aINS appears to provide a multilevel integrated meta-representation of the state of the entire body (Damasio and Carvalho, 2013) integrating body sensations and exteroceptive stimuli into a broader context (Craig, 2009; Farb et al., 2013).

The aINS also integrates information from multiple brain structures and circuits ranging from higher-order cognitions, physiologically driven motivational states, to emotional awareness, which serves to maintain homeostasis (Craig, 2009). Afferent sensory signals are integrated on multiple levels providing a set of interoceptive maps across different body systems (Khalsa et al., 2018). The insular cortex has reciprocal connections to the ACC and can facilitate cognitive control by acting as the switch between the central-executive network and the default mode network DMN (Cole and Schneider, 2007; Sridharan et al., 2008; Menon and Uddin, 2010). This system appears to function as a “cognitive control network” and forms a highly interconnected core system for task-dependent control of goal directed behavior and sensory processing (Brass and Haggard, 2007; Cole and Schneider, 2007; Dosenbach et al., 2007). Sensory signals are sent from the body to the interoceptive network, specifically the pINS to the aINS and then from the aINS to the prefrontal cortex (PFC) bringing subtle, body awareness into conscious awareness (Farb et al., 2015).

The internal state of the body along with the top-down processes leads to a homeostatic emotion made up of a feeling dimension represented in the aINS and a motivation dimension represented in the ACC (Craig, 2009). The interoceptive network, specifically the aINS, plays a vital role in human awareness, which includes body ownership and decision making (Craig, 2009), the generation of the perceptual choice (Thielscher and Pessoa, 2007), the comparisons of feelings in the present moment with those of the past and future (Preuschoff et al., 2008), and the “feeling of knowing,” which plays an important role in meta-memory processing (Kikyo et al., 2002). This capacity can explain why interoceptive predictions that are associated with trauma or anxiety are often distorted (Paulus and Stein, 2006) as the insula is often unusually overactive in individuals who have experienced trauma (Porges, 2007, 2011; Craig, 2009).

Khalsa et al. (2018) highlight that the interoceptive network is closely linked to the nociceptive and affective systems. Craig (2009) related the deep concept of human awareness to interoception and emotional awareness. He argued that all stimuli or sensations that are salient to the individual such as bodily sensations, intentions, emotions, and cognitions are represented by feelings. These feelings are crucial neuropsychological constructs and are the currency of awareness. Zaki et al. (2012) demonstrate that the interoceptive network is not only engaged in interoceptive processing but also highly engaged in emotional processing and that “emotional experience is intimately tied to information about internal bodily states” (p. 498). Several studies have shown that individuals who are more aware of their body report more intense emotional experiences than those who are less aware (Barrett et al., 2004; Critchley et al., 2004; Pollatos et al., 2007). Emotional experiences and intuition may be associated with individual differences in the ability to both generate and perceive subtle bodily changes (Dunn et al., 2010). Studies have shown that learning to read bodily signals through contemplative training can enhance the ability to understand one’s emotions (Villemure et al., 2014; Bornemann et al., 2015; Bornemann and Singer, 2016; Garcia-Cordero et al., 2017).

Finally, it is believed that consciousness of “self” seems to depend on awareness of the body (Cameron, 2001; Ehrsson, 2007; Haselager et al., 2012; Seth, 2013; Farb et al., 2015; Schmalzl et al., 2015). Interoceptive awareness grounds us in the direct experience of the present moment (Damasio, 2003; Farb et al., 2007). Cameron (2001) wrote: “The body and subjective awareness of the body, including visceral awareness, instantiates the ‘self’ and provides the intermediary by which the nervous system interacts with the external world” (p. 708). “In this view, the neural basis for awareness is the neural representation of the physiological condition of the body, and the homeostatic neural construct for a feeling from the body is the foundation for the encoding of all feelings” (Craig, 2009 p. 66). Interestingly, there are specialized neurons called Von Economo Neurons (VENs), which are primarily found in the interoceptive network (Craig, 2009) and the Enteric Nervous System (Mayer, 2011). Von Economo Neurons are believed to enable fast, integrated representations of the emotional moment and behavior and underlie the joint activity between the aINS and ACC (Craig, 2009). Frontotemporal dementia is strongly associated with degeneration of the insular cortex, specifically the VENs found therein. This degeneration was correlated with a loss of self-conscious behavior and emotional awareness of self and others (Seeley et al., 2007).

The insula and interoceptive network are implicated not just with all subjective feelings but also with attention, cognitive choices, intentions, interoceptive and exteroceptive sensory awareness and body movements, visual image of the self, subjective expectations, and subjective trustworthiness of other individuals (see Craig, 2009), among other functions. Craig argues further that no other brain region is activated in all of these tasks, and the only common feature is that they engage the awareness of the subject. Thus, the insula and interoceptive network appear to be the cortical structures and networks that engender human awareness in the present moment – a defining feature in the mindfulness literature. Craig (2002) argued that IA is an evolving and developing capacity that may have an adaptive function by differentiating and refining internal feelings. Interoceptive functions not only enable bodily processes that are necessary for survival such as homeostasis and social interaction (Damasio, 2000; Porges, 2011) but may enable an athlete or musician to enhance their performance by “listening to their body” (Craig, 2002). More accurate perception of body signals may facilitate more adaptive responses to pain (Villemure et al., 2014), healthier eating habits (Herbert et al., 2013), improvements in social relationships (Porges, 2011; Gibson, 2014), better stress management (Bornemann et al., 2015), and other adaptive behaviors.

## How Mindfulness Modulates the Insula

Mindfulness has been defined as moment-to-moment awareness, which is cultivated by paying attention in a specific way, in the present moment, as non-reactivity, non-judgmentally, and open-heartedly as possible (Kabat-Zinn, 2011). Some have argued that attention is the most important aspect of mindfulness (Brown and Ryan, 2003), and this form of attention can shape one’s experience and perspective (Shapiro et al., 2006). Two studies specifically have helped illuminate these processes.

Farb et al. (2007) identified two neural circuits/structures that underlie two different forms of self-reference. The first was referred to as a cortical midline, which includes the medial prefrontal cortex (mPFC) and the posterior cingulate (pCC), similar to the DMN described in other studies. When active, this network exerts a type of cognitive activity in which the authors referred to as a narrative focus (NF). Narrative focus calls for cognitive elaboration of mental events and is associated with mind-wandering and rumination. The other network the authors identified was the interoceptive network. This network, by contrast, calls for the inhibition of cognitive elaboration on a mental event in favor of attending the present moment in a broad, open-monitoring sensing state, which they called experiential focus (EF). Experiential focus was characterized as present-centered, sensing what is occurring in one’s thoughts, feelings, and body state (Farb et al., 2007). The participants in the study were asked to engage in NF which activated the cortical midline by reading trait-related adjectives and reflect on what the adjective meant to them as a person. In the EF mode, participants were asked to monitor their moment-to-moment experience in response to those adjectives. The experimental group went through an 8-week mindfulness training (MT) using the Mindfulness-Based Stress Reduction (MBSR) program. The MBSR program involves a sitting meditation, a body-scan meditation, and yoga.

After MT, the participants engaging EF not only deactivated the cortical midline (DMN) and left lateralized network involved in linguistic related processes but also showed to decouple those areas from the interoceptive network. Conversely, MT increased connectivity within the insula and interoceptive network and produced a larger reduction in the medial PFC. Without MT, participants were more likely to activate the cortical midline and left lateralized circuits when engaging in EF. The authors provide evidence for two distinct modes of self-reference both of which are linked to those two distinct neural circuits: “(1) higher order self-reference characterized by neural processes supporting awareness of a self that extends across time and (2) more basic momentary self-reference characterized by neural changes supporting awareness of the psychological present. The latter, represented by evolutionary older neural regions, may represent a return to the neural origins of identity, in which self-awareness in each moment arises from the integration of basic interoceptive and exteroceptive bodily sensory processes” (pp. 319). After MT, there was a gradual shift in self-referential processes toward a more objective, self-detached analysis rather than a traditional affective or subjective self-referential value associated with the DMN.

The second study conducted by Farb et al. (2013) discovered that the control group – participants who were untrained in MBSR – when asked to focus on internal or external sensations revealed a consistent pattern of dorsomedial prefrontal cortex (DMPFC) activation. The experimental group, those who were trained in MBSR, showed reduced activity in the DMPFC and increased connectivity between the posterior and anterior insula leading to greater insula activation. The authors theorize that MT may simultaneously reduce DMPFC recruitment, which has been linked to the DMN in the cortical midline structures (Farb et al., 2007) and strengthen negative DMPFC/insular connectivity. Thus, DMPFC deactivation during IA indicates a departure from mind-wandering to a more expansive and diffuse form of sensory attention.

Following MT, the DMPFC demonstrated IA-specific negative connectivity to the interoceptive network. The authors highlight that when the DMPFC is active for executive functions, it has also been shown to suppress or limit unintentional interoceptive suppression. In other words, when focusing attention on the body, the connection between the DMPFC and the interoceptive network is absent during executive function, though these network activations appear to be context dependent (Farb et al., 2013). In addition to dampened DMPFC activity, there was increased connectivity between the posterior, mid, and anterior portions of the insula. Both trained and untrained populations in MT were able to activate interoceptive signals from the posterior to anterior insula when focusing on their breath; the difference was that MT participants appeared to possess this increased connectivity by default. This enhanced connectivity provides the individual with a consistent “online” representation of interoceptive awareness even during exteroceptive stimuli or higher order cognitions (Farb et al., 2013).

The authors found that participants who received MT also became more mindful toward pain perception. The experienced meditators focused more attention on bottom-up nociceptive cues represented in the posterior insula and less on evaluative, top-down processes represented in the DMPFC – which has been associated with cognitive appraisal. Those neural changes were linked to a perceptual change in the experience of pain. Both the experiment and control group found the experience intense, but only MT group found the experience less unpleasant during a state of mindful attention. The authors argue that in the absence of cognitive evaluation or judgment, cognition may be freed to consider alternative interpretations of sensory states creating a meta-cognitive awareness (Farb et al., 2013).

I believe these findings are significant. The DMPFC processes higher-order cognitions and relays that information to the aINS as part of a top-down, evaluative process. Focused attention on the body (i.e., meditation) dampens DMPFC activity causing the aINS to attend more fully to the incoming internal stimuli being sent to the pINS from the body leading to a neuroplasticity change in the posterior, mid, and anterior insula (Damasio and Carvalho, 2013; Farb et al., 2013) while simultaneously decoupling the insula from the DMN and DMPFC (Farb et al., 2007, 2013). A more recent study confirmed this finding (Yeng et al., 2018). Yeng et al. (2018) found that focused attention on external stimuli and internal sensations yielded distinct neural signatures and participants who were able to focus on their breath (interoceptive stimuli) over a longer period of time were able to dampen self-referential processing and the neural correlates associated with that function, including the mPFC and the DMN.

It is reasonable to conclude that focused attention on the body, which induces neuroplasticity changes in the insula, DMPFC, and DMN bringing interoceptive information into conscious awareness while simultaneously dampening down higher-order cognitions, provides the mechanism that can explain the gradual changes identified in the mindfulness literature such as increased body awareness, emotional awareness, improvements in attention, and a change in the perspective of the self (Hölzel et al., 2011). The first purpose of this article is to propose that rather than attributing those changes and benefits to mindfulness per se, it may be more accurate to identify those as increased interoception or IA as a result of the neuroplasticity changes within the insula and interoceptive network.

The insula is associated with greater dispositional mindfulness (Creswell et al., 2007; Murakami et al., 2012; Haase et al., 2016), and studies have consistently shown that mindfulness is linked with increased cortical thickness in the aINS (Farb et al., 2007, 2013, 2015; Paulus et al., 2011; Haase et al., 2016). Friedel et al. (2015) have shown that these neuroplasticity changes are true not only in adults but also in adolescents. The authors argue: “While evidence for anterior insula involvement in adult long-term meditator has been interpreted to indicate an effect of mindfulness meditation on insula structure and function, the current results suggest that structural development of the anterior insula may contribute to the development of dispositional mindfulness” (pp. 67). This is a reasonable conclusion as a number of contemplative practices, such as yoga or yoga-based practices (YBPs; Froeliger et al., 2012a; Villemure et al., 2014; Schmalzl et al., 2015), tai chi (Kerr et al., 2008), and all types of meditation (see Fox and Cahn, 2017), have shown to modulate the insula and enhance IA by simply attending to the body without specifically attending to the body in a traditionally mindful way (i.e., non-judgmental, non-reactive, open-hearted).

Given that dispositional mindfulness is believed to enhance self-regulation (Brown and Ryan, 2003; Lakey et al., 2007) and that self-regulation is linked to the interoceptive network – which mindfulness deliberately cultivates, Friedel et al. (2015) suggest a more vital role for the insula in interoception and the maintenance of self-regulation, which is a defining feature in much of the mindfulness literature. Self-regulation may provide a distinct construct with a measurable neurobiological imprint within the insula (Friedel et al., 2015).

Several studies have indicated that YBP may promote a more “mindful” approach to emotionally based stimuli (Gard et al., 2012; Froeliger et al., 2012b) and pain tolerance (Villemure et al., 2014). That is, yoga, like mindfulness, may alter attentional style toward emotional and nociceptive stimuli without trying to cognitively restructure one’s experience (Schmalzl et al., 2015). For example, like Farb et al. (2013), Villemure et al.’s (2014) study demonstrated that participants learned over time to focus on modulating breath or attending to the nociceptive cues in a “mindful” or non-reactive way as compared to controls who would ignore or distract themselves from the pain. Other studies have found that slow, rhythmic breathing also reduced negative affect toward pain stimuli (Zautra et al., 2010; Zeidan et al., 2015). Gibson (2014) found similar results when participants practiced a “non-mindful,” body-centered focused breathing and body scan technique. That is, participants exhibited greater dispositional mindfulness, even though they were simply taught to control their breath and focus on body sensations. These “mindful” adaptations appear to be a consequence of the neuroplasticity changes in the interoceptive system, and Schmalzl et al. (2015) argue that future studies should directly compare YBP with standard mindfulness practices to further clarify these distinctions.

Body-centered contemplative practices have shown to modulate the IA network and alter interoceptive processing by increasing bottom-up integration providing a more accurate representation of what is happening in the body rather than attempting to alter body sensation to fit top-down expectations (Farb et al., 2015). It is being argued here that the practice of attending to bodily sensations strengthens connectivity within the insula and interoceptive network. This network has been found to be neurological correlate of interoceptive, nociceptive, emotional, and all subjective awareness (Craig, 2009; Khalsa et al., 2018). There is also empirical evidence that this network drives attentional states by altering attentional style, which, in some, lead to a broad, open-monitoring sensing state focused in the present moment and enhancing awareness of one’s thoughts, feelings, and body state (Farb et al., 2007, 2013, 2015). These changes could explain the gradual shift identified in the mindfulness literature from a FA or OM technique. Bornemann and Singer (2016) postulate that it might not matter which contemplative practice one practices, what may be the most important factor is sustained, consistent effort.

The body plays a central role in a number of contemplative practices, including mindfulness. There are also a number of body-oriented healing and therapy modalities such as Feldenkrais (Buchanan and Ulrich, 2001), bioenergetics (Lowen, 2006), focusing (Gendlin, 1996), Hakomi (Kurtz, 2007), breath therapy (Mehling, 2001), somatic experiencing (Payne et al., 2015), and many others. In the Buddhist philosophy, the first pillar to develop mindfulness is to develop a sense of the body, which includes an awareness of momentary sensation while distinguishing sensation from conceptual thought (Buddhaghosa, 2010).

The ability to attend to interoceptive sensations can also disrupt automatic, habitual responses to stress and enable more adaptive, regulatory strategies, which can enable one to more easily focus on select interoceptive signals integrating them into a broader contextual representation (Farb et al., 2013). There is evidence that the ability to maintain attention on conditioned responses appears to be necessary for successful extinction of conditioned responses (Hölzel et al., 2011). Kerr et al. (2013) proposed that body-focused attention can enhance attentional processing and attentional control by altering brain frequencies within the 7–14 Hz alpha rhythm that is believed to play a crucial role in regulating signal-to-noise ratio for sensory cortices and throughout the cortex (Kerr et al., 2011). This frequency allows interoceptive information to be more efficiently filtered and prioritized throughout the brain (Schmalzl et al., 2018). Increased access to interoceptive information may provide a richer set of data from which to investigate the relationship between habitual response, interoceptive sensations, and cognitive experiences (Farb et al., 2015).

As interoceptive signals inform emotional experience, contemplative practices may illuminate how afferent signals trigger bodily responses, emotional reactions, and cognitive appraisals, which can then be used to develop adaptive strategies to regulate stress and enhance well-being (Farb et al., 2015). Interoception is foundational to emotional experience, and thus, IA becomes the basis for engaging emotional processing.

## The Body Stores Trauma

Increasing IA in a healthy population has shown to provide a number of benefits; however, increasing IA without compensatory measures may be detrimental to others. It is not uncommon for a person with a history of abuse or extreme trauma to be overcome with anxiety to the point that it is impossible to experience or notice positive body sensations. Farb et al. (2015) argue that there may be an absence of interoceptive information and that fears or anxiety may take on a quality of rumination that is often associated with those clinical conditions. This might explain how rumination can dominate attention. Other research has shown that activation of the anterior insula is associated with anxiety level (Terasawa et al., 2013; Mallorquí-Bagué et al., 2014). As Porges (2011) points out, traumatized individuals have an abnormally active IA network, specifically the insular cortex. The insula has been shown to have a meta-memory function in comparing feelings in the present moment with those of the past and anticipation of the future (Kikyo et al., 2002; Preuschoff et al., 2008). Thus, attending to the body could activate that network potentially eliciting previous mental models or conditioned responses related to the trauma that are too aversive to be accepted or controlled.

Meditation, mindfulness, or any body-focused practice can trigger autonomic hyperarousal, perceptual disturbances, traumatic memory re-experiencing, and even psychosis (see Van Dam et al., 2017 for review). Relaxation-induced panic or anxiety may be the most well-documented phenomena with clear relevance to meditation (Van Dam et al., 2017). The autonomic nervous system can be classically conditioned, and attending to the body may simply elicit previous mental models and conditioned responses of past trauma. Therefore, simply enhancing IA without compensatory regulation may be maladaptive for some individuals (Mehling et al., 2012; Farb et al., 2015; Mehling, 2016; Hanley et al., 2017). By contrast, for others, attending to body sensations may transmit a quality of peace and tranquility and can put the individual into a grounded, calm, and present-focused “being-mode” (Kabat-Zinn, 1990).

## Attentional Style

McGilchrist (2012) argues that attention is not just another “function” alongside other cognitive functions. Its ontological status precedes cognitive functions. “The kind of attention we bring to bear on the world changes the nature of the world we attend to, the very nature of the world in which those ‘functions’ would be carried out, and in which those ‘things’ would exist. Attention changes what kind of thing comes into being for us: in that way it changes the world” (p. 28). McGilchrist maintains that attention is intrinsically a relationship, not an objective fact. “It is a ‘howness,’ a something between, an aspect of consciousness itself, not a ‘whatness,’ a thing in itself, an object of consciousness” (p. 29). Attention brings into being a world with its own set of values and perceptions.

Attention regulation is the basis of all meditative techniques and appears to be a prerequisite for the other beneficial mechanisms to take place (Hölzel et al., 2011; Farb et al., 2015). Interoceptive awareness and mindfulness are associated but distinct constructs in mind-body interactions. One challenge is how to measure attentional style. For example, mindfulness does not often distinguish between attention directed to interoceptive sensations, exteroceptive stimuli, or conscious thoughts. This may be significant as several recent studies highlight that different types of attention elicit different neural signatures (Fox and Cahn, 2017; Yeng et al., 2018). Much of the foci of the interoception literature is on bodily experiences but fails to distinguish between different attention styles (Mehling, 2016). For instance, the term interoceptive sensibility (IS) is ambiguous and does not differentiate from anxiety or hypervigilant style versus a more mindful and open style toward interoceptive sensations (Hanley et al., 2017). Training individuals to focus solely on interoceptive sensations does not automatically imbue participants with knowledge on how to alter attentional style or mental habits commonly employed to avoid unpleasant sensations when those emerge (Bornemann et al., 2015; Mehling, 2016).

Dispositional mindfulness may promote more adaptive interoceptive attentional styles and enhance or illuminate discriminative capacities related to various bodily sensations (Mehling et al., 2009; Hanley et al., 2017). That is, mindfulness may provide a safe focal point from which one can view distressing signals from the body. As Hanley et al. (2017) argue: “awareness of bodily sensations and the evaluative or regulatory tendencies applied to such sensations are important determinants of emotional health” (p. 5). This type of attentional style may require a conscious effort to become more open, accepting, and non-judgmental for some individuals to be able to experience the body as safe and trustworthy. Being mindfully observant and non-judgmental toward body sensations seems to be closely linked to sustained attentional control on body sensations, which is connected to awareness between bodily sensations and emotional states. This mindful state may promote a tendency to listen to the body for insight and as a form of regulating emotions (Kurtz, 2007; Bornemann et al., 2015; Mehling, 2016; Hanley et al., 2017). Individuals who experience their bodies as safe are more likely to report greater psychological well-being. Those who are able to use body awareness for adaptive behavior can be shaped by mindful attentional styles (Tsur et al., 2016; Hanley et al., 2017).

To be clear, in a healthy population, simply attending to the body from a variety of contemplative practices appears to increase IA with its associated benefits (Gibson, 2014; Villemure et al., 2014; Bornemann et al., 2015; Schmalzl et al., 2015), but increasing IA in a clinical population can be problematic. Hanley et al. (2017) point out, if one can bring a mindful attentional style and dampen behavioral reactivity to the discomforting or anxiety provoking sensations, then a refined awareness of those sensations and emotions may reduce hyperarousal. Hanley et al. (2017) argue, non-reactive does not mean non-responsive, rather it indicates a selective attention with less affective interference. Craig (2009) highlights that studies investigating goal directed attention show activation in the aINS and ACC. More specifically, the target awareness is engendered by the aINS, and control of the directed effort is engendered by the ACC. Being able to accept body sensations without judgment may reduce the emotional impact of unpleasant ones. This capacity may enable one to “listen” to those emotion-related sensations that are central to insight and decision making rather than being “overrun” by them (Mehling et al., 2017).

Hanley et al. (2017) found that more observant and less reactive individuals may be more likely to attend to body sensations and use those for regulation purposes. If the body is experienced as safe and comforting, sustained attention on body was found to be easier and intuitive. The authors posit that using body sensations as a form of self-regulation can be interpreted as emotional anchors providing a sense of calm stability as trusting and attentional regulation were significant predictors of psychological well-being.

## A Contemporary Perspective

According to both Eastern traditions and recent contemplative research, increased awareness to bodily information can provide a rich array of information. Rather than trying to anchor scientific findings in a set of abstract and varied practices and techniques (e.g., mindfulness), it may prove more useful to anchor those findings in the body. Focused attention on internal signals is necessary to develop IA and to recognize emotions. Research shows that the body and IA inform and shape all of our subjective experience (Damasio, 1994; Craig, 2009). Therefore, if we grant the primacy to the body as the source in shaping subjective, psychological experience, then the body provides a concrete grounding from which we can investigate mindfulness, interoception, and other contemplative practices and mind-body constructs. Mindfulness and interoception are distinct, yet related constructs and one of the ways in which they differ is in attention. The second purpose of this article is to propose attentional style as a means to not only clarify some of the similarities between interoception, mindfulness, and other contemplative practices, but also help tease apart some of the important differences.

There are a number of ways in which one can attend to the body, and each style can reveal different insights and understandings (Kabat-Zinn, 1990; Gendlin, 1996; Kurtz, 2007; Gibson, 2014). Different types of attention can function like different focal points, each revealing certain dimensions that may be unavailable from other attentional styles. For example, the attentional style in a focused attention (FA) meditation differs from the attentional style of an open monitoring (OM) or mindfulness meditation, and those two differ from the attentional style of a loving-kindness meditation. All meditations with their varying attentional styles produce different psychological, neurological, and functional effects (Fox et al., 2014; Fox and Cahn, 2017). Attentional style also seems to alter how body signals are integrated with higher, top-down processes (Hölzel et al., 2011; Farb et al., 2015; Mehling, 2016). Therefore, if we can anchor these findings in and grant primacy to the body rather than a set of abstract techniques, we can use attentional style to help reveal how various contemplative practices can cultivate and provide unique insight and understanding that may be unavailable from other traditions. This framework may provide a perspective that can add clarity to the mindfulness and meditation literature.

## Focused Attention as an Attentional Style: Rhythmic Breathing

In an attempt to organize and structure the various meditative practices, some models have suggested basic categories that include FA and OM techniques. These techniques all prescribe different attentional styles. I will describe several below to support my thesis. Focused attention on the body has shown to increase interoception and produce neuroplasticity effects in the interoceptive network. This can explain a number of benefits identified in the literature as discussed throughout this article. One of the prominent FA techniques is breath regulation. Several studies have found that focus on the breath activates the interoceptive network (Lutz et al., 2008; Fox et al., 2016). Controlled, rhythmic breathing provides a stream of sensory information that has an affective component (Davenport and Vovk, 2009; Haase et al., 2016). Modulating breath also increases vagal tone, dampens sympathetic nervous system activity (Calabrese et al., 2000; Brown and Gerbarg, 2005; Schmalzl et al., 2015), promotes parasympathetic dominance (Sovik, 2000; Brown and Gerbarg, 2005), and facilitates stress regulation and cognitive control (Sharma, 2014). Increased vagal tone is also correlated with increased heart rate variability HRV. Greater resting HRV is associated with a number of health benefits including greater sustained attention, working memory, and motor control (Thayer et al., 2009), which in turn seems to generate feelings of calm, peace, satisfaction, and a greater connection to others (Farb et al., 2015).

Increased vagal tone mediates vagal afferent information sent to the insula and interoceptive network, which are involved in self-regulatory processes and may promote synchronization of cortical areas (Calabrese et al., 2000; Schmalzl et al., 2015). It is also believed that one’s ability to redirect attention to the body can help improve focused attention, regulate stress, and gain an insight into their emotional-motivational state (Bornemann et al., 2015; Fissler et al., 2016). The interoceptive network plays a key role in disengaging from self-referential thought, which is particularly important from a clinical perspective as this ability can cultivate emotional and cognitive flexibility (Baer, 2003; Yeng et al., 2018) and is a central component for most mindfulness traditions (Mehling et al., 2012; Bornemann et al., 2015; de Jong et al., 2016). Attending to the body anchors the mind in the present and away from rumination (Farb et al., 2015). In short, focused attention on the body activates the insula and interoceptive network, which increases IA with its associated functions. Increased IA is closely related to changes in perspective on the self, which is consistent with the Buddhist philosophy and the mindfulness literature (Hölzel et al., 2011).

## Focused Attention: Body Scan

The body scan is another type of meditation included in the FA meditation literature. The body scan typically consists of focusing attention sequentially on various parts of the body. To be clear, one can bring a “mindful” attention style to this practice, but the body scan *per se* does not necessarily prescribe a mindful approach. Carmody and Baer (2008) found that the body scan was significantly related to the mindfulness constituents of observing and non-reactivity as well as improvements in psychological well-being and decrease in anxiety. In this particular study, the authors implemented the MBSR program, which specifically highlights a mindful attentional style when attending to body sensations. A body scan intervention regularly increases IA, which can be beneficial for some, detrimental for others. As noted above, increased IA does not automatically generate feelings of tranquility but can often trigger a variety of uncomfortable sensations. Bringing a mindful attentional style to a body scan meditation can be an adaptive approach to this practice.

One qualitative study investigated the differences between a focused breathing meditation and a body scan meditation (Gibson, 2014). Even though both meditations are considered to be a FA meditation (Raffone and Srinivasan, 2010; Fissler et al., 2016), both seem to produce different effects. The participants were asked to describe the differences between the two after a month of practice. For some, the body scan helped the participants relax and feel rejuvenated. Others experienced discomfort and anxiety. One participant wrote: “Sensation exercise [body scan] leaves me aware of nothing but my anxiety or nervousness. I don’t never experience full relaxation or any benefit. I am only aware of how uncomfortable I am while performing it or after it” (p. 164) While another wrote: “I love the release I feel in my muscles, letting go of tension in the body scanning meditation is so amazing. It puts me into this more transcendent mental state. I feel so alive and I love it” (p. 163). Most participants identified the focused breathing meditation with relaxation and focus while identifying the body scan with an increased bodily awareness. The increased bodily awareness and insight typically led to a variety of behavioral adaptations.

## Focused Attention: Yoga-Based Practices

Carmody and Baer (2008) also found that yoga was significantly associated with changes in mindfulness, specifically observing, acting with awareness, non-judging, and non-reactivity as well as improvements in well-being, perceived stress levels, and several types of psychological symptoms. Interestingly, yoga was the only formal practice in that MBSR training that was significantly related to the non-judging facet of mindfulness. The authors suggested that the body scan meditation prepared the participants to be more mindful and, hence, derive more benefit from the yoga practice. It is also plausible, and this is the central thesis of this article, that these practices (e.g., body scan meditation and yoga) increase IA as evidenced by the neuroplasticity changes in the insula, interoceptive network (Villemure et al., 2014; Schmalzl et al., 2015) leading to the outcomes identified in the extant literature.

## Open Monitoring or Mindfulness

The ability to be open, accepting, and non-judgmentally notice various sensations without getting carried away can prove useful, especially to those within a clinical population. For instance, when individuals with a history of abuse or trauma recognize sensory signals from the body, which triggers an emotional response, the space created by a mindful attentional style allows the individual to maintain awareness of their bodies instead of dissociating from those sensations into habitual or conditioned responses (Farb et al., 2015). Over time, participants can discover that their bodies can be a helpful resource rather than a source of threat that should be avoided (Farb et al., 2015). Those bodily sensations, which were previously encoded as a threat, can now be integrated into broader states of consciousness that can help the person develop a new self-schema and sense of safety in the world (Price, 2005; Porges, 2011; Farb et al., 2015).

This type of attentional style can help many develop a meta-cognitive awareness that enables individuals to disidentify or disengage from their own emotions and bodily feelings allowing the individual to simply observe or witness them (Mehling, 2016). It is a recognition that one is having an experience rather than “being” the experience. Bodily sensations can simply be experienced rather than transform the experience into a self-defining attribute. Open monitoring or mindfulness meditation affords an important focal point from which we can investigate our embodied self that may be unavailable from FA techniques, especially when FA techniques elicit discomforting sensations.

## Focusing as an Attentional Style

Focusing is another body-centered meditation developed to access what Gendlin (1996) described as a bodily knowing or a “felt sense.” Focusing consists of a series of steps that are similar yet distinct from mindfulness. Rather than attending to the body in an open-monitoring, accepting, non-reactive attitude, focusing attends to the body with a particular type of attention meant to unveil unconscious bodily knowing. This attentional style is not focused on mere body sensation or getting in touch with one’s feelings, or simply observing them, but rather it is focused on connecting to a broader, deeper physical sense of meaning that is done by asking particular questions and waiting for the body to respond. This attentional style can open an aperture to a deeper dimension where sensations reveal a rich array of information all within a broader state of consciousness (Gendlin, 1996).

## Conclusion

With the increasing interest and study of mindfulness in Western science, it is important to be precise in how it is defined as a construct and to differentiate it from other meditative techniques. Van Dam et al. (2017) urged scientists, practitioners, and the media alike to move away from the broad use of the term mindfulness and more clearly specify exactly what practices and processes are being taught. For instance, both FA and OM meditations require different attentional styles and techniques, yet both have been clumped together and have been referred to as a mindfulness meditation. The MBSR program, for instance, consists of multicomponent treatments and employs both FA meditative techniques (body scan and yoga) and an OM or mindfulness technique (sitting meditation). Yet, all three of these interventions require different attentional styles, and it is unclear what role “mindfulness” may play (Hölzel et al., 2011).

One of the central components in the mindfulness and meditation literature is that all meditative processes modulate the insula and interoceptive network (Fox and Cahn, 2017). Given that mindfulness training modulates the insula and interoceptive network, which provides a distinct experiential mode of self-reference in which thoughts, feelings, and bodily sensations are viewed as an integral part of the self and those feelings are experienced more as transient mental events that can be observed in a distant, objective sense rather than self-identifying sensations (Farb et al., 2007) provides an explanatory framework that can advance our understanding of mindfulness, interoception, and other mind-body practices. The insula and interoceptive network are implicated in all bodily, emotional, and subjective feelings and play a central role in attention, intention, and other cognitive functions (see Craig, 2009). Friedel et al. (2015) argue that the interoception plays a central role in the maintenance of self-regulation, which is a distinct feature in the mindfulness literature and provides a measurable neurobiological imprint within the insula (Friedel et al., 2015). Kabat-Zinn (1990) argued that focusing attention on internal sensations cultivates non-judgmental moment-to-moment awareness that permeates into daily life. Hölzel et al. (2011) in a summative article relay how mindfulness shapes (1) attention regulation, (2) body awareness, (3) emotion regulation, and (4) change in perspective on the self. Yet, all of those effects have been linked to the interoceptive network as the neural correlates of those functions. Therefore, it may be more accurate to attribute those effects to increased IA.

Interoceptive signals do not map easily onto objective measures (Mehling, 2016). This can be problematic in Western science because interoceptive processing can be noisy and ambiguous (Petersen et al., 2014). To objectively measure IA as a complex multidimensional capacity is an ongoing process. I have argued here that attentional style may help clarify some of the inconsistent and conflicting findings in the mindfulness and interoception literature. In a healthy population, simply attending to the body has shown to promote a number of benefits. For others, attending to the body can elicit discomforting sensations to where the participant cannot feel comfortable in his or her body. If we grant the body a central role in this process, then attentional style can function like a focal point from which to investigate complex interoceptive signals. Each meditation with its given attentional style/focal point can reveal different perspectives or dimensions of our embodied nature. Craig (2002), and others argue that consciousness of the “self” is believed to depend on awareness of the body. The nature of attention to our body changes the very experience with and perception of it, which, inevitably, changes ourselves.

## Author Contributions

The author confirms being the sole contributor of this work and has approved it for publication.

### Conflict of Interest Statement

The author declares that the research was conducted in the absence of any commercial or financial relationships that could be construed as a potential conflict ofinterest.
